# Protective Effects of Orexin A in a Murine Model of Cisplatin-Induced Acute Kidney Injury

**DOI:** 10.3390/jcm11237196

**Published:** 2022-12-03

**Authors:** Jungmin Jo, Jung-Yeon Kim, Jaechan Leem

**Affiliations:** 1 Division of Hematology-Oncology, Department of Internal Medicine, Ewha Womans University Mokdong Hospital, Seoul 07985, Republic of Korea; 10003kj@ewha.ac.kr; 2 Department of Immunology, School of Medicine, Daegu Catholic University, Daegu 42472, Republic of Korea; jy1118@cu.ac.kr

**Keywords:** orexin A, cisplatin, acute kidney injury, oxidative stress, apoptosis, inflammation

## Abstract

Cisplatin is a chemotherapeutic agent widely used in the treatment of various cancers, but its application is often limited due to complications such as acute kidney injury (AKI). Orexins are hypothalamic neuropeptides that modulate the sleep-wake cycle, neuroendocrine function, and the autonomic nervous system. Emerging evidence suggests that orexin A (OXA) has anti-inflammatory and neuroprotective effects in animal models of neuroinflammatory diseases of the central nervous system. However, the effect of OXA on kidney diseases has not been examined. Here, we investigated whether OXA has a protective effect in a murine model of cisplatin-induced AKI. Intraperitoneal administration of OXA ameliorated renal dysfunction, and histological abnormalities in mice injected with cisplatin. OXA inhibited cisplatin-induced oxidative stress through the modulation of prooxidant and antioxidant enzymes. This peptide reduced apoptotic cell death by inhibiting the p53-mediated pathway in mice injected with cisplatin. OXA also alleviated cisplatin-induced cytokine production and macrophage infiltration into injured kidneys. Taken together, these results showed that OXA ameliorates cisplatin-induced AKI via antioxidant, anti-apoptotic, and anti-inflammatory actions. This peptide could be a potential therapeutic agent for cisplatin-induced AKI.

## 1. Introduction

The kidneys are vital organs that play a major role in maintaining homeostatic regulation of the body’s water and ion balance [1]. They are susceptible to various forms of acute injury due to their role as the major eliminator of exogenous drugs and toxins. Acute kidney injury (AKI) is defined by a sudden decrease in kidney function and results from various types of insults, such as decreased renal perfusion and exposure to nephrotoxins [1]. Among nephrotoxic drugs, cisplatin is a chemotherapy drug used to treat many types of cancer [2]. However, the major drawback of cisplatin treatment is that it often leads to treatment failure due to serious side effects, including nephrotoxicity [2,3,4]. Thus, the development of new pharmacological agents for cisplatin-induced AKI has great clinical significance.

Orexins (orexin A and orexin B) are hypothalamic neuropeptides that regulate the sleep-wake cycle [5]. The activity of orexin neurons increases during wakefulness and decreases during sleep. The loss of orexin neurons in humans has been known to be associated with narcolepsy, which is a chronic sleep disorder characterized by excessive daytime sleepiness [5]. Orexins also play an important role in the regulation of the neuroendocrine and autonomic nervous systems [5,6]. Orexins act on two G-protein-coupled receptors, orexin receptor type 1 and type 2 [7]. Orexin receptors are present in the central nervous system (CNS) and many peripheral tissues [8]. Currently, orexin receptor antagonists are used for the treatment of insomnia [5]. In addition, accumulating evidence suggests that the administration of orexin A (OXA) exerts anti-inflammatory and neuroprotective effects in animal models of neuroinflammatory diseases of the CNS [9,10,11,12,13]. Furthermore, OXA also exhibited anti-inflammatory action in endotoxin shock [14] and ulcerative colitis [15]. However, the effect of OXA on peripheral tissue inflammation remains largely unknown. Here, we investigated the potential effect of OXA on cisplatin-induced AKI and explored the underlying mechanism.

## 2. Materials and Methods

### 2.1. Animal Experiments

Seven-week-old male C57BL/6 mice were purchased from HyoSung Science (Daegu, Republic of Korea). Before starting experiments, the mice were acclimated for 1 week under 20–24 °C on a 12/12 h light/dark cycle. Animal experiments were approved by the Institutional Animal Care and Use Committee of the Daegu Catholic University Medical Center (DCIAFCR-180312-20-Y). The mice were randomly grouped into four groups (*n* = 8 in each group): (1) control (Con) group; (2) OXA group; (3) Cisplatin (CP) group; (4) cisplatin plus OXA (CP + OXA) group. The CP group and the CP + OXA group were injected intraperitoneally with cisplatin [15 mg/kg in 0.9% saline; Cayman Chemical, Ann Arbor, MI, USA]. The OXA group and the CP + OXA group were given an intraperitoneal injection of OXA (1 μmol/kg in phosphate-buffered saline (PBS); Tocris Bioscience, Bristol, UK) daily for 4 consecutive days, starting from 1 day prior to 0.9% saline or cisplatin injection. An equal volume of PBS was injected intraperitoneally into the CP group. The doses of cisplatin and OXA were chosen based on previous studies [16,17,18,19]. At 72 h after cisplatin injection, the mice were sacrificed.

### 2.2. Determination of Creatinine, Blood Urea Nitrogen (BUN), and Cytokine Levels

Serum levels of creatinine and BUN were analyzed using an autoanalyzer (Hitachi, Osaka, Japan). Serum and renal concentrations of tumor necrosis factor-α (TNF-α), interleukin-6 (IL-6), and IL-1β were determined using ELISA kits (R&D Systems, Minneapolis, MN, USA) following the manufacturer’s protocols. 

### 2.3. Histological Analysis, Immunohistochemical (IHC) Staining, and Immunofluorescent (IF) Staining

Kidney tissues were fixed in 10% formalin, dehydrated, and embedded in paraffin for hematoxylin and eosin (H&E) and periodic acid-Schiff (PAS). The degree of tubular injury was scored based on the percentage of injured tubules: 0, 0%; 1, ≤10%; 2, 11–25%; 3, 26–45%; 4, 46–75%; and 5, 76–100% [20,21]. IHC staining was conducted using antibodies against neutrophil gelatinase-associated lipocalin (NGAL; 1:100; ab70287; Abcam, Cambridge, MA, USA), 4-hydroxynonenal (4-HNE; 1:100; ab48506; Abcam, Cambridge, MA, USA) or galectin-3 (1:100; ab2785; Abcam, Cambridge, MA, USA). Then, the sections reacted with HRP-conjugated secondary antibodies. The FITC-labeled lotus tetragonolobus lectin (LTL; 1:100; FL-1321; Vector Laboratories, Burlingame, CA, USA) was used to detect the brush border of proximal tubules. Nuclei were counterstained with DAPI. Quantification of positive staining for NGAL, 4-HNE, or LTL was analyzed using an image-analyzing software (IMT i-Solution, Coquitlam, BC, Canada) in 10 random fields per sample. The number of galectin-3-positive cells was counted in 10 random fields per sample.

### 2.4. Western Blot Analysis

Total protein was extracted from tissues using RIPA lysis buffer (Cayman Chemical, Ann Arbor, MI, USA). Electrophoresis was conducted to separate the proteins on nitrocellulose membranes. After blocking, the membranes were reacted with antibodies against NADPH oxidase 4 (NOX4; 1:1000; NB110-58849; Novus Biologicals, Littleton, CO, USA), cleaved caspase-3 (1:1000; #9661; Cell Signaling Technology, Danvers, MA, USA), cleaved poly(ADP-ribose) polymerase-1 (cleaved PARP-1; 1:1000; #9544; Cell Signaling Technology, Danvers, MA, USA), p53 (1:1000; #2524; Cell Signaling Technology, Danvers, MA, USA), Bax (1:1000; sc-7480; Santa Cruz Biotechnology, Santa Cruz, CA, USA) or glyceraldehyde-3-phosphate dehydrogenase (GAPDH; 1:3000; #5174; Cell Signaling Technology, Danvers, MA, USA). Then, the membranes were incubated with HRP-conjugated secondary antibodies. The blots were visualized using the iBright CL1500 Imaging System (Thermo Fisher Scientific, Waltham, MA, USA) and enhanced chemiluminescence reagents (Thermo Fisher Scientific, Waltham, MA, USA). Relative protein levels were quantified using ImageJ software version 1.53j (National Institute of Health, Bethesda, MA, USA). GAPDH was used as an internal control.

### 2.5. Real-Time Reverse Transcription-Polymerase Chain Reaction (RT-PCR)

Total RNA was extracted from tissues using TRIzol reagent (Sigma-Aldrich, St. Louis, MO, USA). The reverse transcription of extracted RNA was conducted for cDNA synthesis. Then, Real-time RT-PCR was performed using the specific primers (Table 1) in the Thermal Cycler Dice Real Time System III (TaKaRa, Tokyo, Japan). Relative expression was calculated using 2^−ΔΔCT^ method, using GAPDH as an internal control.

### 2.6. Assessment of Oxidative Stress and Antioxidant Enzyme Activities

Malondialdehyde (MDA) and 8-hydroxy-2’-deoxyguanosine (8-OHdG) levels were analyzed using the MDA assay kit (Sigma-Aldrich, St. Louis, MO, USA) and the 8-OHdG assay kit (Abcam, Cambridge, MA, USA), respectively. Reduced glutathione (GSH) and oxidized glutathione (GSSG) levels were determined using the GSH detection kit (Enzo Life Sciences, Farmingdale, NY, USA). Activities of catalase and superoxide dismutase (SOD) were measured using commercial kits (Invitrogen, Carlsbad, CA, USA). All analyses were performed following the manufacturers’ instructions.

### 2.7. TdT-Mediated dUTP Nick End Labeling (TUNEL) Staining

Apoptosis was detected in tissues using a TUNEL assay kit (Roche Diagnostics, Indianapolis, IN, USA) following the manufacturer′s protocol. Briefly, the sections were deparaffinized, permeabilized, and incubated in the TUNEL reaction mixture. Positive cells were counted in 10 random fields per sample.

### 2.8. Statistical Analysis

Data were expressed as the mean ± SEM. Statistical significance was assessed by one-way analysis of variance (ANOVA) with Bonferroni’s multiple comparison tests using Prism version 5 (GraphPad Software, San Diego, CA, USA). A *p*-value less than 0.05 was considered significant.

## 3. Results

### 3.1. OXA Ameliorated Functional and Structural Kidney Damage Caused by Cisplatin

**Figure 1:**
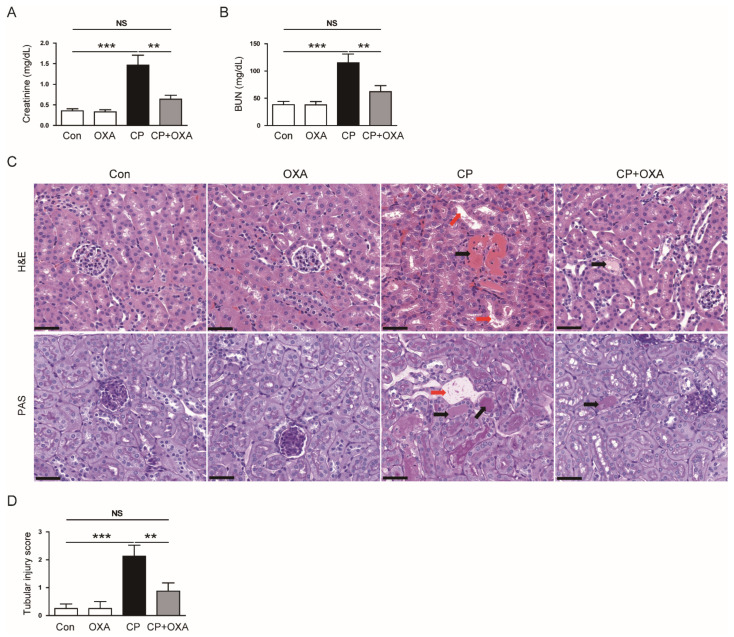
Effects of orexin A (OXA) on renal dysfunction and histological changes in mice injected with cisplatin. (**A**) Serum creatinine levels. (**B**) Blood urea nitrogen (BUN) levels. (**C**) Hematoxylin and eosin (H&E) and periodic acid-Schiff (PAS) staining of kidney sections. Scale bar = 40 μm. Red arrows indicate tubular dilatation. Black arrows indicate cast formation. (**D**) Tubular injury score. ** *p* < 0.01 and *** *p* < 0.001. NS, not significant.

Mice injected with cisplatin had elevated serum creatinine and BUN concentrations (Creatinine: Con, 0.36 ± 0.05 mg/dL vs. CP, 1.46 ± 0.24 mg/dL, *p* < 0.001; BUN: Con, 38.3 ± 6.1 mg/dL vs. CP, 115.0 ± 16.1 mg/dL, *p* < 0.001) (Figure 1A,B). However, OXA significantly reduced the elevated levels of both markers of kidney function (Creatinine: CP, 1.46 ± 0.24 mg/dL vs. CP + OXA, 0.64 ± 0.09 mg/dL, *p* < 0.01; BUN: CP, 115.0 ± 16.1 mg/dL vs. CP + OXA, 62.0 ± 11.4 mg/dL, *p* < 0.01) (Figure 1A,B). Next, the effect of OXA on the histopathological changes induced by cisplatin was analyzed. Histological examination revealed that mice injected with cisplatin exhibited histological abnormalities such as tubular dilation and cast formation (Figure 1C,D). These changes were significantly alleviated by OXA (Tubular injury score: CP, 2.1 ± 0.4 vs. CP + OXA, 0.9 ± 0.3, *p* < 0.05) (Figure 1C,D).

**Figure 2:**
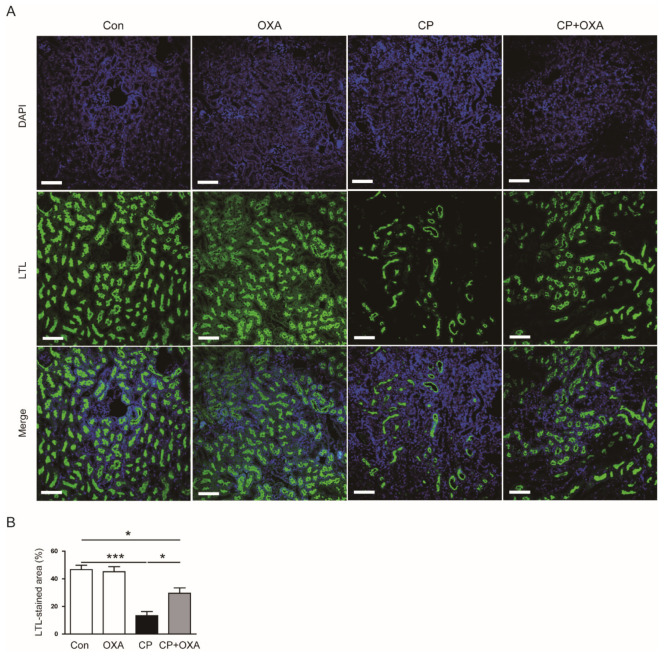
Effect of OXA on the brush border loss of the proximal tubules in mice injected with cisplatin. (**A**) Lotus tetragonolobus lectin (LTL) staining of kidney sections. Scale bar = 100 μm. (**B**) Percentages of LTL-positive area. * *p* < 0.05 and *** *p* < 0.001.

Next, we performed IF staining for LTL to visualize the brush borders of the proximal tubules [22,23]. Mice injected with cisplatin had largely reduced areas of LTL staining (Con, 46.6 ± 3.2% vs. CP, 13.3 ± 3.1%, *p* < 0.001) (Figure 2A,B). However, OXA significantly attenuated the brush border loss (CP, 13.3 ± 3.1% vs. CP + OXA, 29.5 ± 3.9%, *p* < 0.05) (Figure 2A,B).

**Figure 3:**
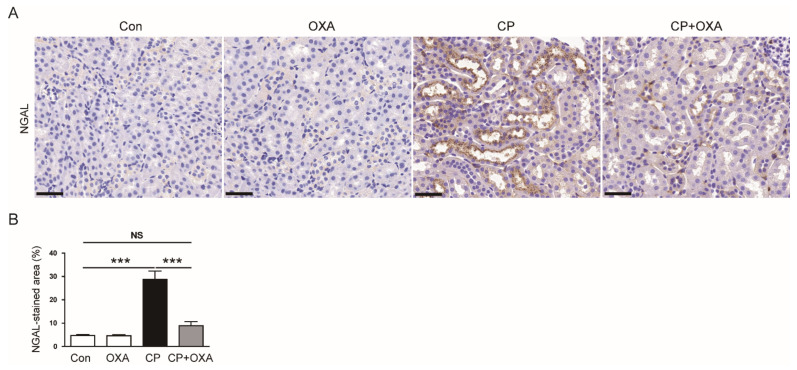
Effect of OXA on neutrophil gelatinase-associated lipocalin (NGAL) expression in mice injected with cisplatin. (**A**) Immunohistochemical (IHC) staining of kidney sections for NGAL. Scale bar = 40 μm. (**B**) Percentages of NGAL-positive area. *** *p* < 0.001. NS, not significant.

In addition, IHC staining for NGAL, a tubular injury marker [24,25], revealed that increased expression of NGAL after cisplatin injection was significantly reduced by OXA (CP, 28.8 ± 3.5% vs. CP + OXA, 9.0 ± 1.7%, *p* < 0.001) (Figure 3A,B).

### 3.2. OXA Attenuated Cisplatin-Induced Oxidative Damage

**Figure 4:**
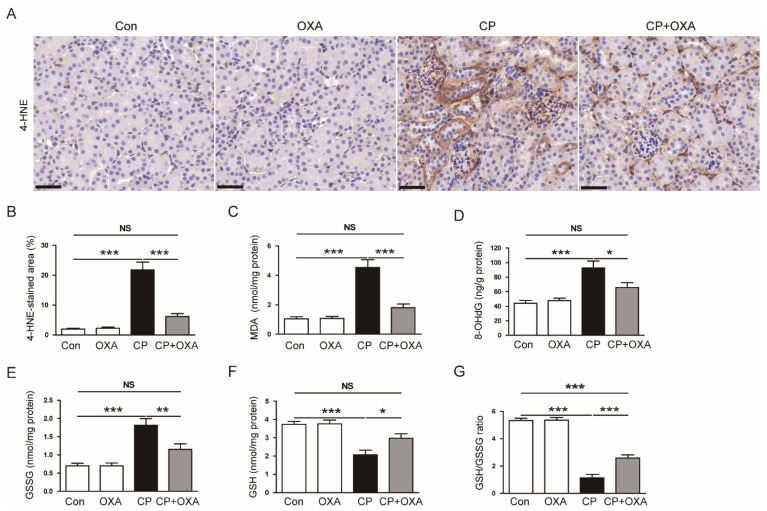
Effect of OXA on oxidative stress in mice injected with cisplatin. (**A**) IHC staining of kidney sections for 4-hydroxynonenal (4-HNE). Scale bar = 40 μm. (**B**) Percentages of 4-HNE-positive area. (**C**) Renal malondialdehyde (MDA) levels. (**D**) Renal 8-hydroxy-2’-deoxyguanosine (8-OHdG) levels. (**E**) Renal oxidized glutathione (GSSG) levels. (**F**) Renal reduced glutathione (GSH) levels. (**G**) GSH/GSSG ratio. * *p* < 0.05, ** *p* < 0.01 and *** *p* < 0.001. NS, not significant.

Oxidative stress plays a crucial role in cisplatin-induced AKI [2,3,4]. OXA has been shown to exert potent antioxidant effects [26,27]. To examine the effect of OXA on oxidative stress in mice injected with cisplatin, kidney sections were reacted with an antibody against the lipid peroxidation product 4-HNE [28]. Mice injected with cisplatin exhibited a marked increase in the percentage of 4-HNE-positive area in kidneys (Con, 2.0 ± 0.3% vs. CP, 21.8 ± 2.7%, *p* < 0.001) (Figure 4A,B). However, OXA significantly reduced the 4-HNE stained area (CP, 21.8 ± 2.7% vs. CP + OXA, 6.2 ± 1.0%, *p* < 0.001) (Figure 4A,B). Renal levels of MDA, another lipid peroxidation product [29], were also reduced by OXA (CP, 4.5 ± 0.5 nmol/mg protein vs. CP + OXA, 1.8 ± 0.3 nmol/mg protein, *p* < 0.001) (Figure 4C). In addition, OXA reduced renal levels of 8-OHdG, a product of oxidative DNA damage [30] (CP, 92.6 ± 9.6 ng/g protein vs. CP + OXA, 65.9 ± 6.5 ng/g protein, *p* < 0.05) (Figure 4D). OXA also increased GSH levels (CP, 2.1 ± 0.3 nmol/mg protein vs. CP + OXA, 3.0 ± 0.2 nmol/mg protein, *p* < 0.01) and GSH/GSSG ratio (CP, 1.1 ± 0.2 vs. CP + OXA, 2.6 ± 0.2, *p* < 0.001), while decreasing GSSG levels (CP, 1.8 ± 0.2 nmol/mg protein vs. CP + OXA, 1.2 ± 0.2 nmol/mg protein, *p* < 0.05) (Figure 4E–G).

**Figure 5:**
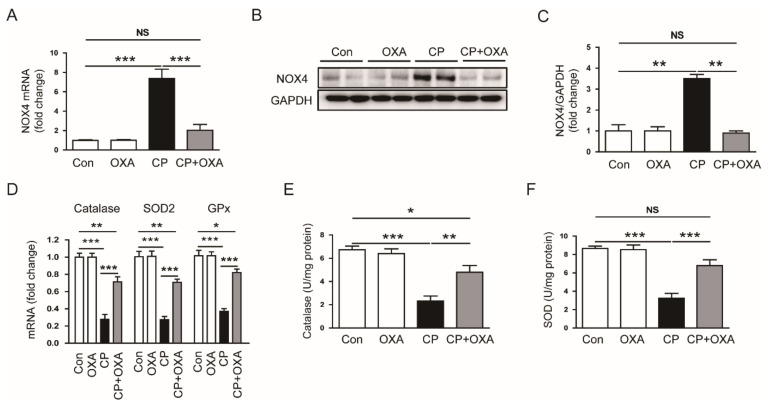
Effect of OXA on prooxidant and antioxidant enzymes in mice injected with cisplatin. (**A**) Renal nicotinamide adenine dinucleotide phosphate oxidase 4 (NOX4) mRNA levels. (**B**) Western blotting of NOX4. (**C**) Quantification of protein expression of NOX4. (**D**) Renal catalase and superoxide dismutase 2 (SOD2) mRNA levels. (**E**) Catalase activity. (**F**) SOD activity. * *p* < 0.05, ** *p* < 0.01 and *** *p* < 0.001. NS, not significant.

NOX4 is a major prooxidant enzyme that plays an important role in ROS production in the kidney [31]. Recent studies have shown that cisplatin injection promotes tissue damage by increasing renal expression of NOX4 [32,33]. Therefore, we next examined the effect of OXA on NOX4 expression in the kidney. Cisplatin injection increased mRNA (Con, 1.0 ± 0.1 vs. CP, 7.4 ± 0.9, *p* < 0.001) and protein (Con, 1.0 ± 0.3 vs. CP, 3.5 ± 0.2, *p* < 0.01) levels of NOX4, which was significantly attenuated by OXA (NOX4 mRNA: CP, 7.4 ± 0.9 vs. CP + OXA, 2.0 ± 0.6, *p* < 0.001; NOX4 protein: CP, 3.5 ± 0.2 vs. CP + OXA, 0.9 ± 0.1, *p* < 0.01) (Figure 5A–C). We also examined the expression of antioxidant enzymes. Mice injected with cisplatin exhibited reduced mRNA levels of catalase, SOD2, and glutathione peroxidase (GPx) compared to control mice (catalase: Con, 1.00 ± 0.05 vs. CP, 0.28 ± 0.06, *p* < 0.001; SOD2: Con, 1.00 ± 0.06 vs. CP, 0.27 ± 0.04, *p* < 0.001; GPx: Con, 1.02 ± 0.06 vs. CP, 0.37 ± 0.03, *p* < 0.001) (Figure 5D). Cisplatin injection also inhibited the activity of catalase (Con, 6.7 ± 0.3 U/mg protein vs. CP, 2.3 ± 0.4 U/mg protein, *p* < 0.001) (Figure 5E) and SOD (Con, 8.7 ± 0.2 U/mg protein vs. CP, 3.2 ± 0.5 U/mg protein, *p* < 0.001) (Figure 5F). However, these changes were significantly reversed by OXA (catalase mRNA: CP, 0.28 ± 0.06 vs. CP + OXA, 0.71 ± 0.06, *p* < 0.01; SOD2 mRNA: CP, 0.27 ± 0.04 vs. CP + OXA, 0.71 ± 0.04, *p* < 0.001; GPx mRNA: CP, 0.37 ± 0.03 vs. CP + OXA, 0.82 ± 0.04, *p* < 0.001; catalase activity: CP, 2.3 ± 0.4 U/mg protein vs. CP + OXA, 4.8 ± 0.6 U/mg protein, *p* < 0.01; SOD activity: CP, 3.2 ± 0.5 U/mg protein vs. CP + OXA, 6.8 ± 0.6 U/mg protein, *p* < 0.001) (Figure 5D–F). 

### 3.3. OXA Inhibited Cisplatin-Induced Apoptosis

**Figure 6:**
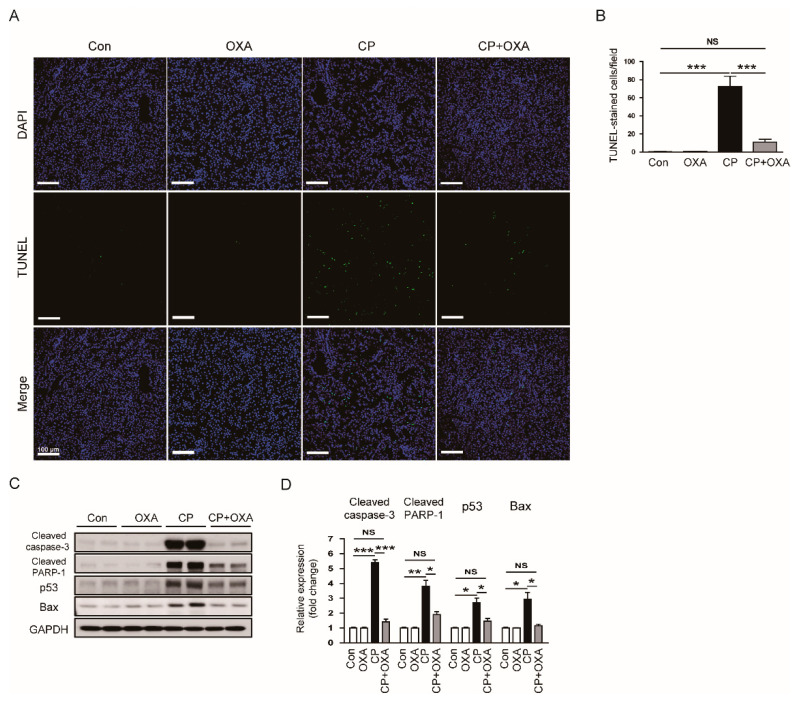
Effect of OXA on apoptotic cell death in mice injected with cisplatin. (**A**) TdT-mediated dUTP nick end labeling (TUNEL) staining on kidney sections. Scale bar = 50 μm. (**B**) Number of TUNEL-positive cells. (**C**) Western blotting of cleaved caspase-3, cleaved poly(ADP-ribose) polymerase-1 (cleaved PARP-1), p53, and Bax. (**D**) Quantification of protein expression of cleaved caspase-3, cleaved PARP-1, p53, and Bax. * *p* < 0.05, ** *p* < 0.01 and *** *p* < 0.001. NS, not significant.

Cisplatin can induce apoptotic death of tubular epithelial cells [34,35]. Tubular cell apoptosis also plays an important role in cisplatin-induced AKI [2,3,4]. Therefore, we next performed TUNEL staining on kidney sections to detect apoptotic cells. Mice injected with cisplatin showed a marked increase in the number of TUNEL-positive cells (Con, 0.4 ± 0.3 vs. CP, 72.5 ± 11.4, *p* < 0.001) (Figure 6A,B). However, OXA reduced the number of apoptotic cells (CP, 72.5 ± 11.4 vs. CP + OXA, 10.9 ± 3.1, *p* < 0.001) (Figure 6A,B). Protein expression of cleaved caspase-3, cleaved PARP-1, p53 and BAX was also reduced by OXA (cleaved caspase-3: CP, 5.4 ± 0.2 vs. CP + OXA, 1.4 ± 0.2, *p* < 0.05; cleaved PARP-1: CP, 3.8 ± 0.4 vs. CP + OXA, 1.9 ± 0.2, *p* < 0.05; p53: CP, 2.5 ± 0.3 vs. CP + OXA, 1.7 ± 0.2, *p* < 0.05; Bax: CP, 2.5 ± 0.5 vs. CP + OXA, 1.2 ± 0.1, *p* < 0.05) (Figure 6C,D).

### 3.4. OXA Attenuated Cisplatin-Induced Inflammatory Responses

**Figure 7:**
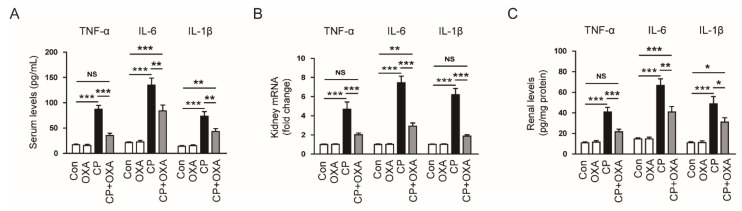
Effect of OXA on serum and renal levels of cytokines in mice injected with cisplatin. (**A**) Serum tumor necrosis factor-α (TNF-α), interleukin-6 (IL-6) and IL-1β levels. (**B**) Renal TNF-α, IL-6 and IL-1β mRNA levels. (**C**) Renal TNF-α, IL-6 and IL-1β protein levels. * *p* < 0.05, ** *p* < 0.01 and *** *p* < 0.001. NS, not significant.

Cisplatin induces cytokine production and secretion from renal tubular epithelial cells and immune cells [2,3,4]. In addition, OXA has been shown to have anti-inflammatory activity [14,15]. Therefore, we next investigated the effect of OXA on cisplatin-induced cytokine production. Mice injected with cisplatin had increased serum levels of TNF-α, IL-6, and IL-1β compared to controls (TNF-α: Con, 16.8 ± 1.5 pg/mL vs. CP, 87.3 ± 0.2 pg/mL, *p* < 0.001; IL-6: Con, 21.4 ± 1.6 pg/mL vs. CP, 134.8 ± 13.8 pg/mL, *p* < 0.001; IL-1β: Con, 73.6 ± 8.9 pg/mL vs. CP, 43.3 ± 5.5 pg/mL, *p* < 0.001). However, OXA significantly reduced the levels of these proinflammatory cytokines (TNF-α: CP, 87.3 ± 0.2 pg/mL vs. CP + OXA, 35.1 ± 4.7 pg/mL, *p* < 0.001; IL-6: CP, 134.8 ± 13.8 pg/mL vs. CP + OXA, 83.9 ± 11.6 pg/mL, *p* < 0.01; IL-1β: CP, 73.6 ± 8.9 pg/mL vs. CP + OXA, 43.3 ± 5.5 pg/mL, *p* < 0.01) (Figure 7A). Renal mRNA (Figure 7B) and protein (Figure 7C) levels of these proinflammatory cytokines were also largely elevated after cisplatin injection but were significantly attenuated by OXA (TNF-α mRNA: CP, 4.7 ± 0.8 vs. CP + OXA, 2.0 ± 0.2, *p* < 0.001; IL-6 mRNA: CP, 7.5 ± 0.7 vs. CP + OXA, 2.9 ± 0.3, *p* < 0.001; IL-1β mRNA: CP, 6.2 ± 0.6 vs. CP + OXA, 1.9 ± 0.1, *p* < 0.001; TNF-α protein: CP, 41.0 ± 0.8 pg/mg protein vs. CP + OXA, 21.6 ± 2.5 pg/mg protein, *p* < 0.001; IL-6: CP, 66.8 ± 6.3 pg/mg protein vs. CP + OXA, 40.9 ± 5.5 pg/mg protein, *p* < 0.01; IL-1β: CP, 48.8 ± 7.2 pg/mg protein vs. CP + OXA, 31.0 ± 4.4 pg/mg protein, *p* < 0.05).

**Figure 8:**
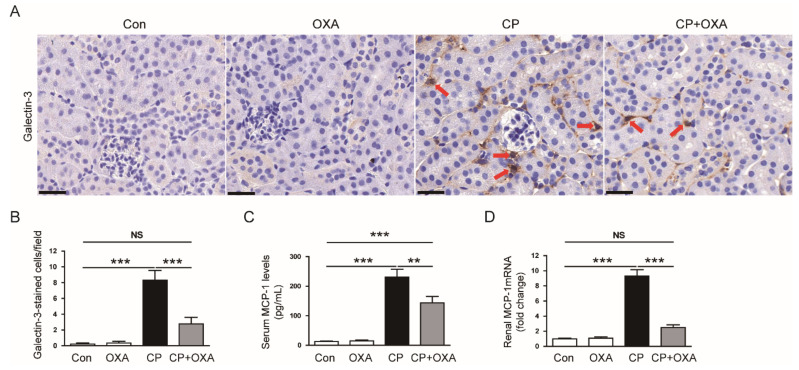
Effect of OXA on macrophage accumulation and monocyte chemoattractant protein-1 (MCP-1) expression in mice injected with cisplatin. (**A**) IHC staining of kidney sections for galectin-3. Red arrows indicate galectin-3-positive cells. Scale bar = 60 μm. (**B**) Number of galaectin-3-positive cells. (**C**) Serum MCP-1 levels. (**D**) Renal MCP-1 mRNA levels. ** *p* < 0.01 and *** *p* < 0.001. NS, not significant.

It has been known that macrophages are infiltrated into injured kidneys after cisplatin injection and play an important pathogenic role in cisplatin-induced AKI [2,3,4]. To evaluate the effect of OXA on macrophage infiltration, kidney sections reacted with an antibody against the macrophage marker galectin-3 [36]. The number of galectin-3-stained cells was increased after cisplatin injection (Con, 0.2 ± 0.1 vs. CP, 8.3 ± 1.2, *p* < 0.001) (Figure 8A,B). However, OXA significantly reduced the number of macrophages (CP, 8.3 ± 1.2 vs. CP + OXA, 2.8 ± 0.8, *p* < 0.001) (Figure 8A,B). In addition, serum (Con, 12.5 ± 1.6 pg/mL vs. CP, 230.8 ± 26.7 pg/mL, *p* < 0.001) (Figure 8C) and renal (Con, 1.0 ± 0.1 vs. CP, 9.3 ± 0.8, *p* < 0.001) (Figure 8D) levels of monocyte chemoattractant protein-1 (MCP-1), a main macrophage-attracting chemokine [16], were increased after cisplatin injection but were significantly decreased by OXA (serum MCP-1: CP, 230.8 ± 26.7 pg/mL vs. CP + OXA, 143.3 ± 21.9 pg/mL, *p* < 0.01; MCP-1 mRNA: CP, 9.3 ± 0.8 vs. CP + OXA, 2.5 ± 0.4, *p* < 0.001).

## 4. Discussion

In the present study, we investigated the effect of OXA on cisplatin-induced AKI in mice. Systemic administration of OXA ameliorated renal dysfunction and histopathological alterations in mice injected with cisplatin. OXA alleviated cisplatin-induced oxidative stress through the regulation of prooxidant and antioxidant enzymes. Apoptotic cell death in mice injected with cisplatin was also attenuated by OXA. This peptide inhibited cytokine production and macrophage infiltration.

Although OXA is known to modulate the sleep-wake cycle, recent studies have shown that this peptide has anti-inflammatory effects in murine models of several inflammatory diseases [9,10,11,12,13,14,15]. In this study, we found that OXA reduced serum levels of creatinine and BUN, established markers of kidney function, in cisplatin-injected mice, suggesting that OXA protects mice from cisplatin-induced renal dysfunction. In addition, OXA attenuated cisplatin-induced structural damage, as evidenced by a decrease in the tubular injury score, an increase in the area of positive staining for LTL, and a decrease in NGAL expression. Because LTL is a marker for the brush borders of the proximal tubules [24,25], a decrease in the area of LTL staining indicates a brush border loss. The inhibitory effect of OXA on the tubular injury was also confirmed by decreased expression of the tubular injury marker NGAL. Collectively, these results demonstrate the protective effect of OXA on cisplatin-induced AKI.

Oxidative stress is a hallmark of cisplatin-induced AKI [2,3,4]. Therefore, we next investigated changes in oxidative stress to study the mechanism for the protective effect of OXA on cisplatin-induced AKI. The amounts of 4-HNE and MDA were measured to assess lipid peroxidation, and the amount of 8-OHdG was evaluated to assess DNA oxidation. As previously reported [29,37], cisplatin injection markedly increased lipid peroxidation and DNA oxidation in mice. Moreover, the GSH/GSSG ratio, an established indicator of oxidative stress [38], was decreased after cisplatin injection. However, OXA significantly inhibited cisplatin-induced oxidative stress. To further evaluate the mechanisms underlying the inhibitory effect of OXA on oxidative stress, we examined the expression or activity of prooxidant and antioxidant enzymes. Cisplatin injection increased NOX4 expression and decreased the expression and activity of catalase, SOD, and GPx. It has been shown that NOX4 is a major source of intracellular reactive oxygen species (ROS) and plays an important role in cisplatin-induced AKI [31,32,33]. In addition, it has been shown that cisplatin injection reduces the expression and activity of several antioxidant enzymes in rodents, exacerbating cisplatin-induced oxidative damage [2,3,4]. However, OXA significantly reversed cisplatin-induced changes in prooxidant and antioxidant enzymes. These findings suggest that OXA inhibits cisplatin-induced oxidative stress through the regulation of prooxidant and antioxidant systems. Consistent with our findings, a recent study showed that OXA inhibited NOX4 expression and ROS production in high glucose-exposed human endothelial cells [26]. Activation of the antioxidant defense system was also observed in OXA-treated human endothelial cells [27].

Tubular cell apoptosis plays an important role in cisplatin-induced AKI [2,3,4]. Previous studies have shown that OXA exerts a neuroprotective effect by inhibiting neuronal apoptosis [39,40,41]. These results prompted us to investigate the effect of OXA on apoptosis in mice injected with cisplatin. In this study, apoptotic cells were identified using the TUNEL staining. As previously reported [38,42], cisplatin injection largely increased the number of TUNEL-positive apoptotic cells in the kidney. This is also confirmed by the activation of the key executioner caspase, caspase-3. However, OXA significantly attenuated cisplatin-induced apoptosis. Accumulating evidence suggests that the p53 protein is a key player in the cisplatin-induced apoptosis of tubular epithelial cells [43]. This protein is a transcription factor that induces apoptosis through transcriptional regulation of apoptosis-related genes such as Bax. We found that cisplatin injection increased p53 and Bax expression but was inhibited by OXA. Altogether, these results suggest that OXA inhibits cisplatin-induced apoptosis by inhibiting the p53-mediated pathway.

Inflammation is the main process in cisplatin-induced AKI [2,3,4]. Cisplatin-induced inflammatory responses are characterized by excessive production of proinflammatory cytokines and infiltration of immune cells [2,3,4]. In this study, increased serum and renal concentrations of TNF-α, IL-6, and IL-1β were significantly reduced by OXA. In line with our findings, a recent study reported that OXA suppressed inflammatory responses in murine models of colitis [15]. Production of proinflammatory cytokines in immune cells and intestinal epithelial cells was inhibited by OXA [15]. We found that this peptide also alleviated macrophage infiltration in cisplatin-injected mice. Among proinflammatory cytokines, TNF-α has been known to play an essential role in cisplatin-induced inflammation [44]. Pharmacological inhibition or genetic ablation of TNF-α attenuated cisplatin-induced AKI [44]. TNF-α stimulates the production of other proinflammatory cytokines and promotes immune cell infiltration [2,3,4]. Macrophages are one of the cellular sources of proinflammatory cytokines, including TNF-α, and exacerbate cisplatin-induced kidney injury [43]. In this study, serum and renal levels of MCP-1 were reduced by OXA. MCP-1 is a major chemokine that plays a key role in macrophage recruitment [16]. Previous studies have reported that cisplatin injection increased macrophage infiltration along with MCP-1 upregulation in rodents [29,45]. Altogether, our findings suggest that OXA attenuates cisplatin-induced inflammation by inhibiting cytokine production and macrophage infiltration.

It has been known that OXA cannot normally penetrate the blood–brain barrier (BBB) [5]. However, recent studies have shown that OXA can penetrate into the CNS due to BBB dysfunction under the condition of systemic inflammation [13,14]. Peripheral administration of OXA ameliorated inflammatory responses and improved survival in mice with endotoxin shock through its central action [14]. Multiple pathways, including the neuroendocrine and autonomic nervous systems, are involved in the mechanisms underlying the beneficial effect of OXA on septic shock through the CNS [14,46]. Uremia can induce disruption of BBB integrity, and thus AKI has been shown to be associated with BBB dysfunction [47]. Therefore, the protective effect of OXA on cisplatin-induced AKI is likely due to the central action of OXA through the neuroendocrine and autonomic nervous systems. However, because orexin receptors are also widely present in peripheral tissues [8], the possibility that OXA may act directly on the kidney cannot be excluded. Future studies will be needed to elucidate the exact mechanism of the protective effect of OXA against kidney diseases.

## 5. Conclusions

In conclusion, our findings demonstrated that OXA has a protective effect on cisplatin-induced AKI by inhibiting oxidative stress, apoptosis, and inflammation. These results suggest that OXA has the potential to be used as a therapeutic agent for cisplatin-induced AKI.

## Figures and Tables

**Table 1:** List of primers.

Gene	Primer Sequence (5′→3′)	Accession No.
NOX4	F: CCCAAGTTCCAAGCTCATTTCC R: TGGTGACAGGTTTGTTGCTCCT	NM_015760
Catalase	F: CAAGTACAACGCTGAGAAGCCTAAG R: CCCTTCGCAGCCATGTG	NM_009804
SOD2	F: AACTCAGGTCGCTCTTCAGC R: CTCCAGCAACTCTCCTTTGG	NM_0136671
GPx	F: GCAATCAGTTCGGACACCAG R: CACCATTCACTTCGCACTTCTC	NM_008160
TNF-α	F: CACAGAAAGCATGATCCGCGACGT R: CGGCAGAGAGGAGGTTGACTTTCT	NM_013693
IL-6	F: TAGTCCTTCCTACCCCAATTTCC R: TTGGTCCTTAGCCACTCCTTC	NM_031168
IL-1β	F: CGCAGCAGCACATCAACAAGAGC R: TGTCCTCATCCTGGAAGGTCCACG	NM_008361
MCP-1	F: TAAAAACCTGGATCGGAACCAA R: GCATTAGCTTCAGATTTACGGGT	NM_011333
GAPDH	F: ACTCCACTCACGGCAAATTC R: TCTCCATGGTGGTGAAGACA	NM_001289726

## Data Availability

The data supporting the findings of this study are available within the article.
