# Correction: Jo et al. Protective Effects of Orexin A in a Murine Model of Cisplatin-Induced Acute Kidney Injury. *J. Clin. Med.* 2022, *11*, 7196

**DOI:** 10.3390/jcm15010120

**Published:** 2025-12-24

**Authors:** Jungmin Jo, Jung-Yeon Kim, Jaechan Leem

**Affiliations:** 1 Division of Hematology-Oncology, Department of Internal Medicine, Ewha Womans University Mokdong Hospital, Seoul 07985, Republic of Korea; 10003kj@ewha.ac.kr; 2 Department of Immunology, School of Medicine, Daegu Catholic University, Daegu 42472, Republic of Korea; jy1118@cu.ac.kr

In the original publication [1], there was a mistake in Figure 6A (OXA group) as published. The incorrect TUNEL image was inadvertently used during figure preparation. The corrected Figure 6 appears below. The authors state that the scientific conclusions are unaffected. This correction was approved by the Academic Editor. The original publication has also been updated.

## Figures and Tables

**Figure 6:**
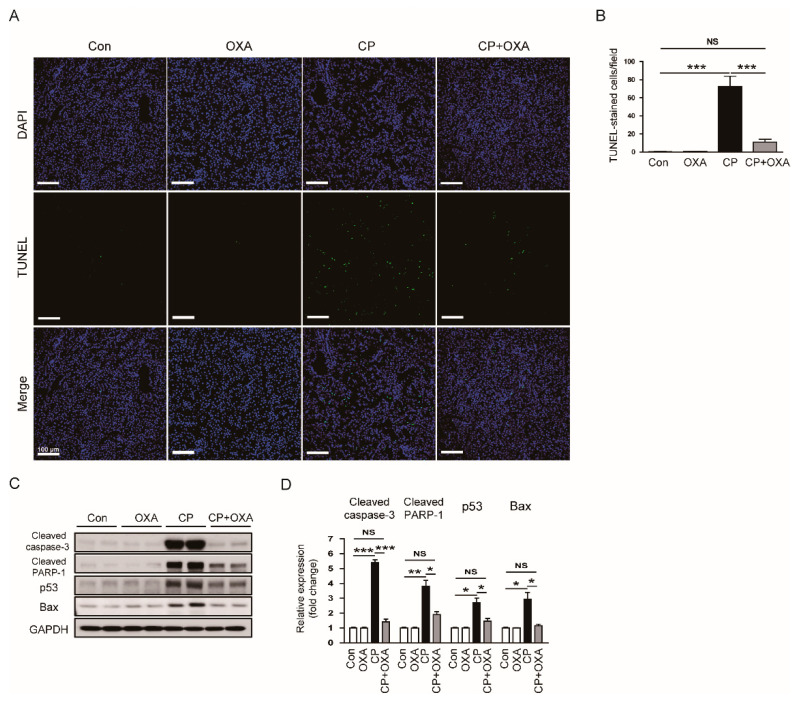
Effect of OXA on apoptotic cell death in mice injected with cisplatin. (**A**) TdT-mediated dUTP nick end labeling (TUNEL) staining on kidney sections. Scale bar = 50 μm. (**B**) Number of TUNEL-positive cells. (**C**) Western blotting of cleaved caspase-3, cleaved poly(ADP-ribose) polymerase-1 (cleaved PARP-1), p53, and Bax. (**D**) Quantification of protein expression of cleaved caspase-3, cleaved PARP-1, p53, and Bax. * *p* < 0.05, ** *p* < 0.01 and *** *p* < 0.001. NS, not significant.
